# Editorial: Contemporary Issues in Defining the Mechanisms of Cognitive Behavior Therapy

**DOI:** 10.3389/fpsyt.2021.755136

**Published:** 2021-09-08

**Authors:** Daniel R. Strunk, Lorenzo Lorenzo-Luaces, Marcus J. H. Huibers, Nikolaos Kazantzis

**Affiliations:** ^1^Department of Psychology, The Ohio State University, Columbus, OH, United States; ^2^Department of Psychological and Brain Sciences, Indiana University Bloomington, Bloomington, IN, United States; ^3^Department of Clinical Psychology, Vrije Universiteit Amsterdam, Amsterdam, Netherlands; ^4^Cognitive Behavior Therapy Research Unit, Melbourne, VIC, Australia; ^5^Beck Institute for Cognitive Behavior Therapy & Research, Philadelphia, PA, United States

**Keywords:** cognitive, behavior, therapy, psychological therapy, process, review, mechanisms

## Introduction

Cognitive behavior therapy (CBT) is an umbrella term that refers to psychological therapies founded on the premise that (a) cognitive and behavioral processes are implicated in the development and maintenance of psychopathology, and (b) those processes are likely to be present during the session and require the therapist to tailor the intervention to best assist the patient (1). CBT includes therapies that focus on attention and other processes of cognition (e.g., acceptance, tolerance), cognitive reappraisal (e.g., decentering, defusion), behavior change (e.g., activation, exposure), as well as emotion coping, and interpersonal skill development (2).

Various CBTs have well-documented efficacy (3, 4) that have led to considerable dissemination efforts (5), but the literature on mechanisms of action is less evolved (6–9). This literature can be classified into three groups: (1) research designed to identify patient features that may serve as prognostic predictors, as well as moderators or mediators of treatment effects (10–12); (2) research focused on generic relational processes in psychotherapy, such as foundational counseling skills, typically assessed as empathy, warmth, positive regard, and agreement on goals and tasks of therapy, comprising the combined construct of working alliance (13, 14); as well as (3) research aiming to examine specific aspects of the delivery of CBT, such as collaborative-empiricism, Socratic dialogue (15) and facilitation of homework assignments (16, 17). These various research efforts each involve methodological challenges including the indexing of patient characteristics and evaluation of therapist competence (18, 19).

In this Research Topic, we sought contributions from leading clinical scientists to contribute empirical, review, and conceptual issues in defining the mechanisms of change in CBT.

## New Empirical Studies of Mechanisms of Change in CBT

Four papers examine in-session processes that might explain therapeutic gains in CBT. First, Lemmens et al. examine the processes that might contribute to sudden gains, large, stable improvements, in cognitive therapy (CT) for depression (20). Though primarily descriptive given the small sample size, they found the largest differences between a pre-gain and a control session emerged for their measures of behavioral changes. Interestingly, the working alliance was consistently high before and after the gain, suggesting it might not be a proximal determinant of sudden gains.

Second, Don et al. examined the temporal associations of the working alliance and therapist adherence in CBT for depression. Initially, they failed to find that the alliance or a therapist-reported adherence item was associated with either subsequent or prior symptom change. In additional analyses, a perceived helpfulness component of the alliance alone was related to prior symptom change. Their work helps to reinforce the importance of carefully timing the assessment of therapy process variables and symptom change when working to understand how treatments achieve their effects.

Third, Feldmann et al. investigated potential mechanisms in CBT for chronic pain within routine inpatient treatment. They found that changes in the specific treatment processes of cognitive restructuring and relaxation were associated with changes in disability and depression. Their work highlights how data from routine care, which often can achieve larger samples than experimental studies, can be used to investigate mechanisms and pathways for improving CBT.

Finally, in a relatively large sample, Beierl et al. examined the relationship between the alliance and outcome in the context of treatment for post-traumatic stress disorder. Therapist and patient-rated alliance at session one were associated with symptom change from baseline to post-treatment. In a more sophisticated cross-lagged panel analysis that focused on prediction of subsequent change across sessions one, three, and five, findings using therapist-rated alliance supported a reciprocal relationship with both the alliance predicting symptom change and symptom change predicting the alliance. Neither of these relationships achieved significance using patient-rated alliance.

## Conceptual Issues in the Search for Mechanisms of Change in CBT

Six papers examine important conceptual issues in the search for mechanisms of change in CBT, with some of these papers also describing newer methodological or analytic strategies. First, Hollon et al. explore how CBTs might fit into an evolutionary account of depression. They suggest that depression and anxiety are coordinated responses that helped individuals prepare to function adaptively in our ancestral past. Depression may have evolved to promote a type of rumination that may help to solve complex social problems. They suggest that, unlike medications which reduce the distress, CBTs may be better suited to facilitating the evolutionary function of depression in helping individuals ruminate more effectively.

Second, Verdonk and Trousselard address the question of how mindfulness works. They introduce the context-updating hypothesis, which posits that mindfulness facilitates optimal adjustment of prior beliefs to the context of present experience and thereby minimizes prediction errors. Prior beliefs are updated more effectively in light of the present context. They explore the clinical applications of this hypothesis and discuss how it could be tested.

Third, Huibers et al. provide an overview of the challenges in developing empirically-driven personalized psychotherapies. They introduce the concept of *personalized causal pathways* that highlight specific paths whereby therapeutic procedures lead to changes in therapeutic processes for patients with specific characteristics. They propose a research agenda that is designed to carefully characterize these pathways to facilitate development of an empirically driven personalization of CBT, including a call for research on both generic and CBT specific in-session processes.

Fourth, Zilcha-Mano and Webb extend prior reviews of processes in CBT and concentrate on studies that propose methodological and statistical approaches for disentangling within-individual (or state-like) effects and between-individual (or trait-like) effects. They effectively highlight the importance of the distinction, noting that it has not been considered in much of the existing psychotherapy process literature. They suggest that between-individual variables might be identified as prognostic or prescriptive factors, whereas within-individual variables are appropriate for evaluating potential active ingredients of treatment. They highlight how research making such distinctions particularly measured well and analyzed appropriately have the potential to substantially advance our understanding of how CBT works.

Fifth, Kaiser et al. report on a network analysis of follow-up effects of internet-delivered CBT. Using network intervention analysis, they were able to identify specific symptoms and aspects of quality of life that were directly impacted by iCBT (compared with care as usual) as well as additional symptoms that appeared to be impacted indirectly because they change following changes in other symptoms. Interestingly, in their study, patients who scored higher on the directly affected items experienced greater benefit from iCBT.

Finally, Southward and Sauer-Zavala discuss sequential multiple assignment randomized trial (SMART) designs as an approach to improving our understanding of treatment mechanisms. They describe their ongoing study in which patients are first randomized to receive the modules of the Unified Protocol in standardized or personalized order. At treatment mid-point, patients are further randomized to either discontinue treatment immediately or continue with the remaining sessions. They propose to test engagement of mechanisms such as distress aversion which might represent an important tailoring variable for whether to end or continue treatment. Their work highlights some innovative ways that experiments can evaluate personalized treatment decisions.

## New Technologies in the Study of Mechanisms of Change in CBT

A final pair of papers examine the use of new technologies to investigate potential mechanisms of CBT. Wu et al. report on their systematic review and meta-analysis of virtual reality-assisted cognitive behavioral therapy (VRCBT) for anxiety. Drawing on data from a handful of available studies, VRCBT outperformed wait-list but did not differ significantly from “standard” CBT. The authors provide a discussion of the potential clinical benefits of VRCBT. Hehlmann et al. provide a preliminary test of ecological momentary assessments using heart rate variability using fitness trackers every 3 min to investigate the role of stress in the process of change in CBT. They highlight how passive assessments might provide much more detailed information about change over time than traditional self-report measures.

## Conclusion

This Research Topic for *Frontiers in Psychiatry* provides a rich sample of contemporary work focused on advancing our understanding of the mechanisms of CBT. With the array of sophisticated methodological and analytic strategies being brought to bear, we are optimistic that a new generation of CBT research will substantially advance our understanding of how CBT achieves its effects and that such an understanding might ultimately be utilized to optimize CBT's benefits.

## Author Contributions

All authors contributed to the ideas and have reviewed this Editorial.

## Conflict of Interest

The authors declare that the research was conducted in the absence of any commercial or financial relationships that could be construed as a potential conflict of interest.

## Publisher's Note

All claims expressed in this article are solely those of the authors and do not necessarily represent those of their affiliated organizations, or those of the publisher, the editors and the reviewers. Any product that may be evaluated in this article, or claim that may be made by its manufacturer, is not guaranteed or endorsed by the publisher.
